# Construction and Validation of Two Hepatocellular Carcinoma-Progression Prognostic Scores Based on Gene Set Variation Analysis

**DOI:** 10.3389/fcell.2022.806989

**Published:** 2022-03-09

**Authors:** Qifan He, Baorui Fan, Peng Du, Yonghai Jin

**Affiliations:** Department of Interventional Radiology, The First Affiliated Hospital of Soochow University, Suzhou, China

**Keywords:** hepatocellular carcinoma, prognostic stratification system, gene set variation analysis, PPI, tumor infiltrating immune cell

## Abstract

**Background:** Liver hepatocellular carcinoma (LIHC) remains a global health challenge with a low early diagnosis rate and high mortality. Therefore, finding new biomarkers for diagnosis and prognosis is still one of the current research priorities.

**Methods:** Based on the variation of gene expression patterns in different stages, the LIHC-development genes (LDGs) were identified by differential expression analysis. Then, prognosis-related LDGs were screened out to construct the LIHC-unfavorable gene set (LUGs) and LIHC-favorable gene set (LFGs). Gene set variation analysis (GSVA) was conducted to build prognostic scoring models based on the LUGs and LFGs. ROC curve analysis and univariate and multivariate Cox regression analysis were carried out to verify the diagnostic and prognostic utility of the two GSVA scores in two independent datasets. Additionally, the key LCGs were identified by the intersection analysis of the PPI network and univariate Cox regression and further evaluated their performance in expression level and prognosis prediction. Single-sample GSEA (ssGSEA) was performed to understand the correlation between the two GSVA enrichment scores and immune activity.

**Result:** With the development of LIHC, 83 LDGs were gradually upregulated and 247 LDGs were gradually downregulated. Combining with LIHC survival analysis, 31 LUGs and 32 LFGs were identified and used to establish the LIHC-unfavorable GSVA score (LUG score) and LIHC-favorable GSVA score (LFG score). ROC curve analysis and univariate/multivariate Cox regression analysis suggested the LUG score and LFG score could be great indicators for the early diagnosis and prognosis prediction. Four genes (ESR1, EHHADH, CYP3A4, and ACADL) were considered as the key LCGs and closely related to good prognosis. The frequency of TP53 mutation and copy number variation (CNV) were high in some LCGs. Low-LFG score patients have active metabolic activity and a more robust immune response. The high-LFG score patients characterized immune activation with the higher infiltration abundance of type I T helper cells, DC, eosinophils, and neutrophils, while the high-LUG score patients characterized immunosuppression with the higher infiltration abundance of type II T helper cells, TRegs, and iDC. The high- and low-LFG score groups differed significantly in immunotherapy response scores, immune checkpoints expression, and IC50 values of common drugs.

**Conclusion:** Overall, the LIHC-progression characteristic genes can be great diagnostic and prognostic signatures and the two GSVA score systems may become promising indices for guiding the tumor treatment of LIHC patients.

## Introduction

Liver cancer is the sixth most common malignant tumor and the fourth most common cause of cancer-related death (Villanueva 2019). Cirrhosis, mostly as a result of hepatitis virus infection or alcohol abuse, is currently considered to be the main cause of liver cancer (Marengo et al., 2016). Liver hepatocellular carcinoma (LIHC) is the most common type of liver cancer. The main treatment strategies for LIHC are surgery, radiotherapy, chemotherapy, and palliative therapies (Llovet et al., 2015). Regrettably, these treatments are less effective in patients with advanced LIHC (Tian et al., 2018). Therefore, it is urgent to explore significant diagnosis and prognosis indicators of LIHC. The wide use of high-throughput sequencing technology in Liver cancer research has revealed many promising targets for the early diagnosis and evaluation of prognosis (Zhang et al., 2017; Calderaro et al., 2019). AFP is the most common biomarker in LIHC for early diagnosis and tumor recurrence surveillance (Pinero et al., 2020). DKK1 has been found highly expressed in HCC tissue and proposed to be a novel HCC biomarker with a very good diagnostic performance (Shen et al., 2012). Higher expression of Glypican-3 was significantly associated with a worse prognosis in LIHC (Xiao et al., 2014). The upregulated expression of TBK1 can enhance tumor immune infiltration and predict the poor prognosis of patients with LIHC (Jiang et al., 2021). Other studies develop prognostic models based on gene sets and carry out validation analyses (Huo et al., 2020; Zhou et al., 2020). Nevertheless, most studies do not take the changes of gene expression patterns in different stages of tumors into account.

In our study, we identified LIHC-unfavorable gene set and LIHC-favorable gene set by integrating gene expression data and corresponding clinical data from TCGA. Gene set variation analysis (GSVA) was used to calculate the enrichment score of LIHC patients and construct two scoring systems. The diagnostic and prognostic capability of two scoring systems were verified in multiple datasets. By integrating the PPI network and univariate Cox regression analysis of all LCGs, ESR1, EHHADH, CYP3A4, and ACADL were determined as the key LCGs. Subsequently, we investigate the expression level and prognostic correlation of the key LDGs in different HCC datasets. Additionally, ssGSEA analysis was used to explore the correlation of the two gene sets with gene alteration and immune infiltration. These findings indicate that the two GSVA scoring systems may become reliable molecular markers and provide targets for the diagnosis and prognosis of LIHC.

## Materials and Methods

### Data Collection

The gene expression data and corresponding clinical features of LIHC patients were downloaded from International Cancer Genome Consortium (ICGC) (Zhang et al., 2019), The Cancer Genome Atlas (TCGA) (Tomczak et al., 2015), and Gene Expression Omnibus (GEO) (Barrett et al., 2013). TCGA LIHC cohorts containing 50 control samples and 374 HCC samples (175 stage I samples, 87 stage II samples, 86 stage III samples, and 26 stage IV samples) were collected for subsequent analyses. In addition, we obtained gene expression array and prognostic information of GSE14520 cohorts (374 HCC samples and 50 control samples) and ICGC LIHC cohorts (212 HCC samples and 177 control samples) as validation sets. The genes with lower expression and samples with no prognostic information were excluded.

### Identification of LIHC-Development Genes

The “normalizeBetweenArrays” function in “limma” R package was performed to background adjustment and quantile normalization. In TCGA datasets, DEGs between normal group and I-IV HCC stage groups were respectively identified utilizing the “limma” package with a fold-change of 1.5 and an adjusted *p*-value of <0.05 (Ritchie et al., 2015). We defined LDG as gradually upregulated DEGs (logFCstage I vs. control < logFCstage II vs. control < logFCstage III vs. control < logFCstage IV vs. control) and downregulated DEGs (logFCstage I vs. control > logFCstage II vs. control > logFCstage III vs. control > logFCstage IV vs. control). Potential functions and enriched pathways of LDGs were further explored by the “clusterProfiler” package (Yu et al., 2012), and *p* < 0.05 was considered as significant.

### Establishment of the LIHC-Progression Gene Set Variation Analysis Score

According to the median expression level of LDGs, all samples were divided into high/low groups and subjected to Kaplan–Meier survival curves analyses, and *p* < 0.05 was considered to be statistically significant. Those LDGs that drastically influenced survival were considered as LIHC-progression characteristic genes (LCGs) and established two prognostic gene sets, including the LIHC-unfavorable gene set (LUGs, related to poor prognosis) and the LIHC-favorable gene set (LFGs, related to good prognosis). Several external microarray datasets (GSE10143, GSE14520, GSE22058, GSE25097, GSE36376, GSE46444, GSE54236, GSE63898, GSE64041, and GSE76427) were performed to validate the differential expression of LCGs between HCC samples and adjacent normal samples.

Gene Set Variation Analysis (GSVA) is a non-parametric, unsupervised algorithm for calculating Normalized Enrichment score (NES) of pathways and functional annotation based on gene expression array, which was extensively utilized in cancer-related studies (Liu et al., 2021a; Liu et al., 2021b; Liu et al., 2021c). Next, we further performed GSVA approach based on the two prognostic gene sets to calculate the NES of each patient as LIHC-unfavorable GSVA score (LUG score) and LIHC-favorable GSVA score (LFG score) using the “GSVA” R package (Hanzelmann et al., 2013). Receiver operating characteristic curve (ROC) analysis was employed to illustrate the diagnostic veracity of two GSVA scores in different HCC cohorts (TCGA, ICGC, and GSE14520). Patients in TCGA, ICGC, and GSE14520 cohorts were divided into high/low-risk groups according to the median scores and subsequently carried out to Kaplan–Meier survival analysis.

### Clinical Correlation Analyses of the LUG Score and LFG Score

To investigate the impact of the two GSVA scores on clinical characteristics, we further explore the relationship of the LUG score and LFG score with other clinical characteristics (age, gender, Child grade, T stage, M stage, N stage, and race). In addition, the univariate Cox regression analysis was employed to evaluate the correlation between prognosis and clinical characteristics, and multivariate Cox regression analysis was applied to analyze the independent prognostic ability of the risk factors.

### Mutation and Immunohistochemistry Analyses of LCGs

To determine the somatic mutations of HCC patients between high- and low-GSVA score groups, the mutation annotation format (MAF) from the TCGA database was generated using the “maftools” R package (Mayakonda et al., 2018). The Human Protein Atlas is a human protein online database including normal and neoplastic tissues (Uhlen et al., 2010). We utilized the Human Protein Atlas web tool to validate the abnormal expression of LCGs between HCC and liver tissues at the protein level.

### Exploration of the Molecular Mechanism

The GSVA method was used to quantify the activity of molecular pathways and find significantly correlated pathways with two GSVA scores. The differences in NES between the high- and low-GSVA scores groups were compared by independent-samples *t*-tests, and *p* < 0.05 was regarded as statistically significant. Gene Ontology (GO) enrichment analysis was performed on the DEGs identified by the “limma” R package between the high- and low-LUG score groups. Gene set enrichment analysis (GSEA) was applied to evaluate the immune response between the high- and low-LUG score groups, and adjusted *p*-value < 0.05 was considered to be different (Subramanian et al., 2005). The gene set “c2.cp.kegg.v6.2.symbols.gmt” and “h.all.v7.2.symbols.gmt” were chosen as the reference gene set.

### Construction of PPI Network and Identification of the Hub LCGs

A PPI network between LDGs was constructed through the Search Tool for the Retrieval of Interacting Genes (STRING) online tool (Szklarczyk et al., 2021). Nodes with interaction scores >0.9 and containing LCGs were imported to the Cytoscape, a software for visualizing complex networks. Additionally, univariate regression analysis was utilized to evaluate the prognostic relevance of the LCGs. The key LCGs were screened out and the selection criteria was the number of adjacent nodes >4 in the network and *p*-value <0.05 in prognostic analysis. The Gene Set Cancer Analysis (GSCA) database integrates comprehensive cancer information from TCGA (Liu et al., 2018). We explored aberrant LCG expression in several types of cancer utilizing the GSCA online tool.

### Comprehensive Analysis of the Key LCGs

The difference in expression level of key LCGs between tumor and normal samples were validated in various datasets using independent-samples *t*-test procedure. And the variation of the key LCGs’ expression pattern as tumor stage increased was verified by the Gene Expression Profiling Interactive Analysis (GEPIA) database (Tang et al., 2017). Simultaneously, the external validation sets (ICGC and GSE14520) were carried out to Kaplan–Meier survival analysis between high- and low-expression groups, which were divided by the median expression value of the key LCGs. In order to further confirm the independent prognostic ability of each key LCGs, we combined the clinical features with the key LCGs to perform multivariate analyses based on TCGA and ICGC data. Furthermore, the GSCA database was employed to investigate the potential mechanism of abnormal expression of key LCGs in multiple aspects, including pathway activity and methylation. Respective co-expression networks of the key LCGs in HCC were achieved through the HCCDB online database (Lian et al., 2018), and then input into Metascape for gene annotation (Zhou et al., 2019).

### Immune Infiltration Analysis and Drug Susceptibility Analysis

Single-sample gene set enrichment analysis (ssGSEA) (Barbie et al., 2009) was conducted to quantify infiltration levels for 24 different immune cell types in TCGA HCC samples (Bindea et al., 2013). The correlation between prognostic signatures and immunocyte infiltration levels was evaluated using the “Pearson” approach. The difference in the distribution of immunocyte infiltrating levels between high- and low-GSVA groups was analyzed by Wilcoxon test. The ESTIMATE score of each sample, comprising StromalScore and ImmuneScore, was calculated using the R package “ESTIMATE” (Yoshihara et al., 2013). The distinction in immune infiltrating level and the ESTIMATE score between high- and low-score groups were analyzed by Wilcoxon test. Immune checkpoint inhibitor (ICI) was an advanced method for activating antitumor immunity (Topalian et al., 2015). Hence, the relationship between the GSVA scores and six common inhibitory checkpoint molecules (CD274, CTLA4, HAVCR2, LAG3, PDCD1, and TIGIT) was assessed to speculate the immunotherapy response targeting ICIs. The Tumor Immune Dysfunction and Exclusion (TIDE) score and Tumor microenvironment evaluation (TME) score are two different computational models for predicting response to immune checkpoint blockade (ICB) (Jiang et al., 2018; Zeng et al., 2021). We uploaded the TCGA transcriptome profiles to the TIDE web and then obtained every patient’s TIDE score, and TME score was computed by “TMEscore” R packages. Moreover, to compare the therapeutic effects of chemotherapeutic drugs in the different score groups, we measure the semi-inhibitory concentration (IC50) values of commonly used chemotherapeutic drugs for LIHC by the “pRRophetic” package (Geeleher et al., 2014).

### Statistical Analyses

All statistical analyses were conducted *via* R software (Version 3.6.7). The Student’s *t*-test was used for statistical comparisons. Spearman’s correlation was applied for the analysis of the correlation. The Benjamini–Hochberg false discovery rate (FDR) method was used for p-value adjustment. Fisher’s test was used to identify the significant GO terms. A *p*-value <0.05 was regarded as statistically significant. The cut-off value of continuous variables, such as gene expression and immune infiltration level, was median.

## Results

### Identification of the LIHC-Development Gene

**FIGURE 1:**
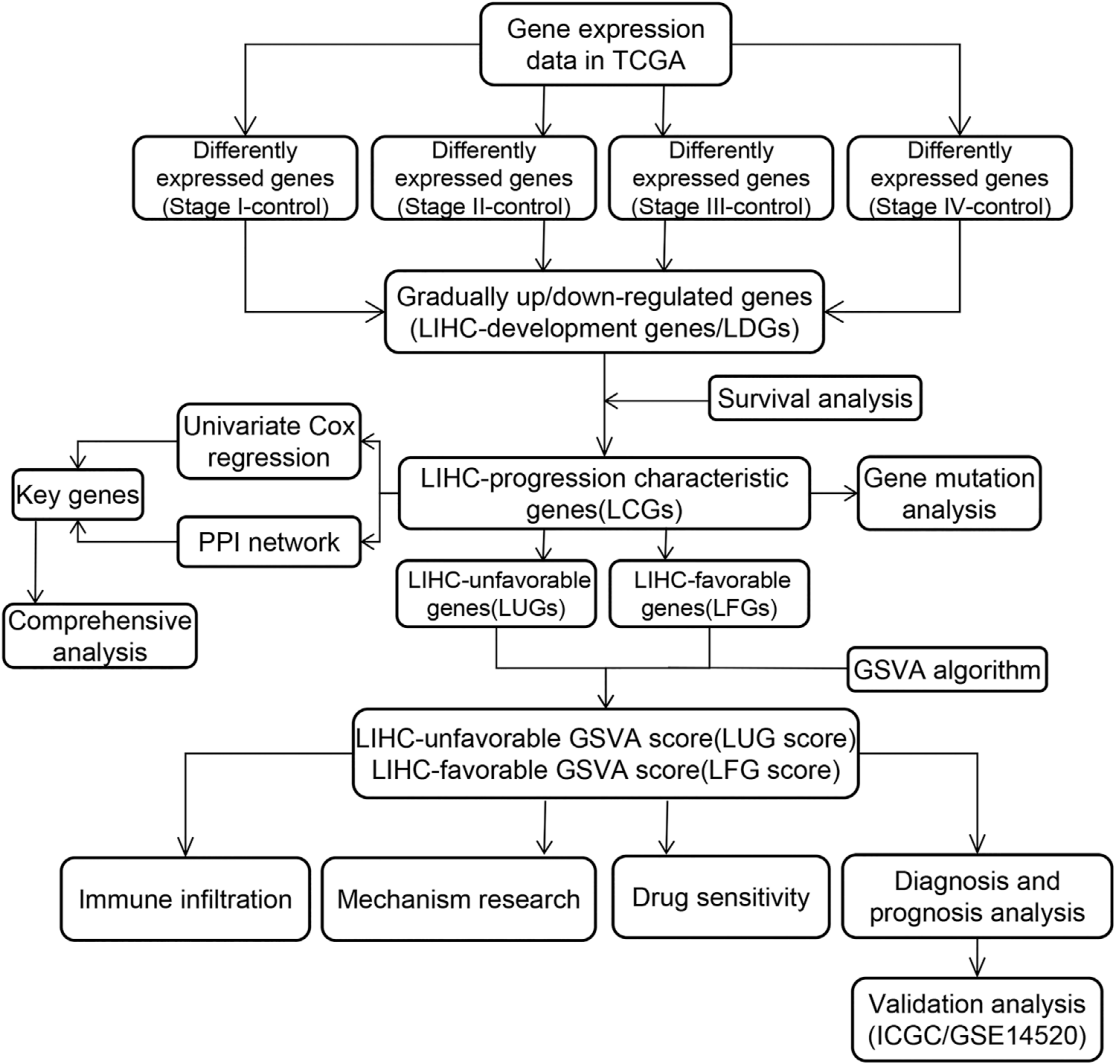
Flow chart of our study.

**FIGURE 2:**
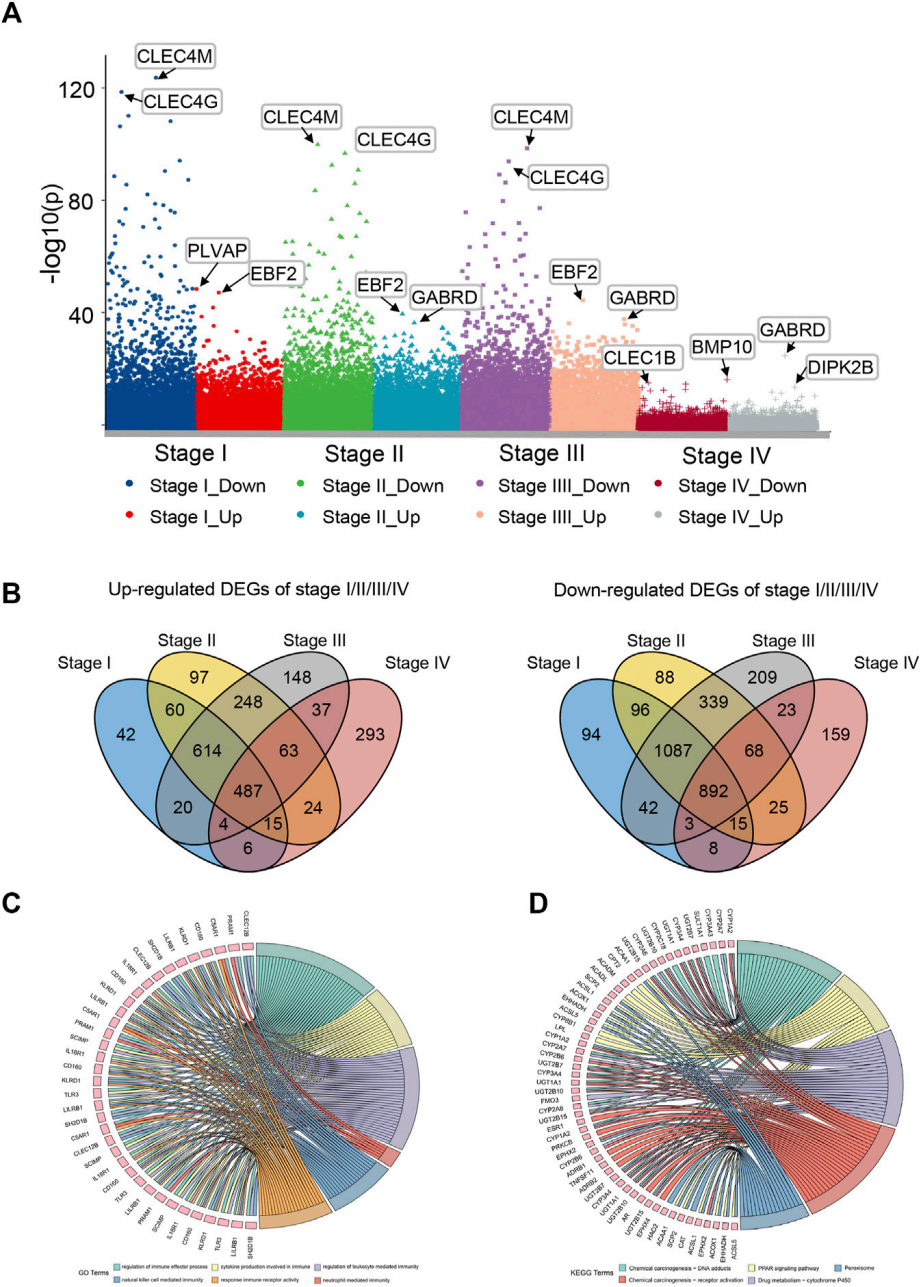
Differential expression gene analysis and functional enrichment analysis. **(A)** Manhattan plot showed differentially expressed genes (DEGs) in different stages of LIHC. **(B)** Venn plot of up/downregulated common DEGs in LIHC stage I–IV. **(C)** GO enrichment analysis of LIHC-development genes. **(D)** KEGG pathway analysis of LIHC-development genes.

The general analysis flow of our study is shown in Figure 1. We screened out a total of 487 common upregulated DEGs, and 892 common downregulated DEGs were identified by the intersection of DEGs between different subgroups (Figure 2B). Among them, 83 DEGs were gradually upregulated and 247 DEGs were gradually downregulated as the stage evolved. These DEGs may have a sustained effect on HCC progression so they are considered as the LIHC-development genes (LDGs). In the result of the GO analysis, the TRGs were mainly associated with the regulation of cell cycle, chromosome segregation, mitotic nuclear division, regulation of inflammatory response and immune effector process, response to drug, and organelle fission (Figure 2F). The result of GO analysis showed that the LDGs were enriched in several immunoregulation ways, such as regulation of the immune effector process, cytokine production involved in immune response, regulation of leukocyte-mediated immunity, and neutrophil-mediated immunity (Figure 2C). As for the KEGG pathway enrichment, the LDGs were mainly associated with the chemical carcinogenesis, PPAR signaling pathway, peroxisome, and drug metabolism of cytochrome P450 (Figure 2D).

### Two Groups of LDGs With Opposite Prognostic Characteristics Were Picked out

**FIGURE 3:**
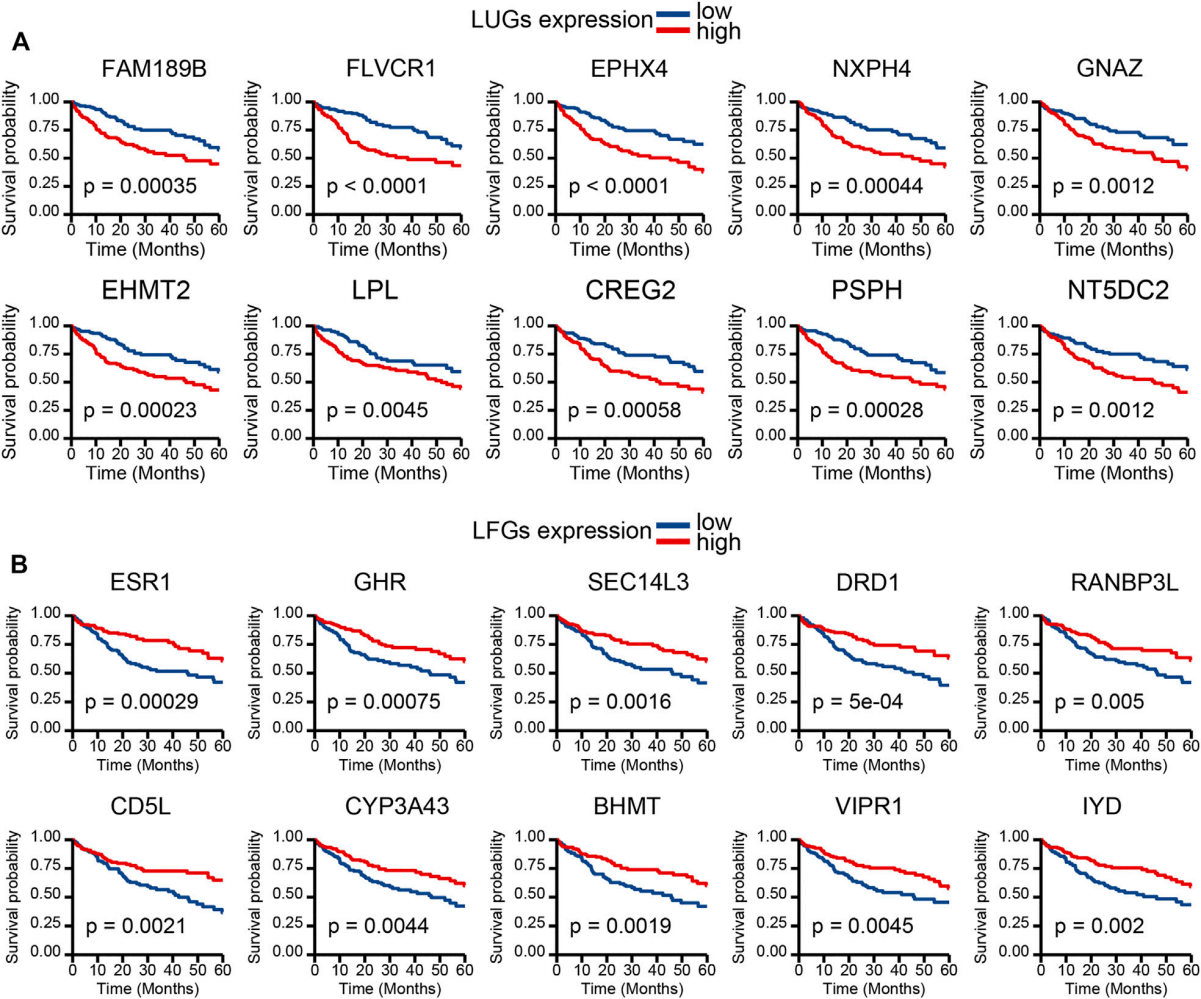
Survival analysis. **(A)** KM survival curve of 10 most significant LIHC-unfavorable genes. **(B)** KM survival curve of 10 most significant LIHC-favorable genes.

Kaplan–Meier (KM) curve analysis discovered that 63 LDGs were prominently associated with clinical outcome and named LIHC-progression characteristic genes (LCGs). Among them, the LIHC-unfavorable gene set (LUGs) contained 31 LCGs related to poor prognosis, while the LIHC-favorable gene set (LFGs) incorporated 32 LCGs linked to good prognosis (Table 1). Kaplan–Meier (KM) curves based on TCGA cohorts of LUGs and LFGs are shown in Figures 3A,B. Additionally, all LCGs were differentially expressed between HCC and adjacent noncancerous tissue in multiple validation datasets from different platforms (Supplementary Figure S1). IHC analyses from HPA database also confirmed aberrant expression of LCGs in tumor tissue (Supplementary Figure S2).

**TABLE 1:** LIHC-unfavorable gene set and LIHC-favorable gene set.

Gene set	Gene symbol
LIHC unfavorable genes	PYGO2, FAM189B, EHMT2, TARBP1, FLVCR1, ADAM15, TIGD1, LAMC1, LPL, EPHX4, EGFL6, CREG2, NXPH4, CEP72, HEY1, PSPH, H4C8, HPDL, GNAZ, NT5DC2, ATP6V0D2, NANOS1, MEX3A, HES2, CHML, GNG4, CYP19A1, ATP8A2, STK39, PNCK, ETV4
LIHC-favorable genes	VIPR1, CPEB3, ESR1, ADRA1A, CD5L, RANBP3L, GHR, HAO2, CYP3A43, ACADL, EPHX2, TERB2, IYD, CCT6B, DMGDH, GBP7, RDH16, SEC14L3, ABCA9, EHHADH, DHRS1, CYP3A4, MOGAT1, BHMT, SLC38A4, PACRG, ACOT12, TTPA, HDC, CYP8B1, HLF, DRD1

HCC, hepatocellular carcinoma; TCGA, the cancer genome atlas; ICGC, international cancer genome consortium; GEO, gene expression omnibus; LDGs, LIHC-development gene; LCGs, LIHC-progression characteristic gene; LUGs, LIHC-unfavorable gene set; LFGs, LIHC-favorable gene set; LUG score, LIHC-unfavorable GSVA score; LFG score, LIHC-favorable GSVA score; DEGs, differential expressed genes; GO, gene ontology; KEGG, Kyoto encyclopedia of genes and genomes; KM, Kaplan–Meier; ROC, receiver operating characteristic; AUC, area under curve; OS, overall survival; GSVA, gene set variation analysis; NES, normalized enrichment score; GSEA, gene set enrichment analysis; ssGSEA, single sample gene set enrichment analysis.

### LIHC-Progression GSVA Score Could Effectively Predict Prognosis for LIHC Patients

**FIGURE 4:**
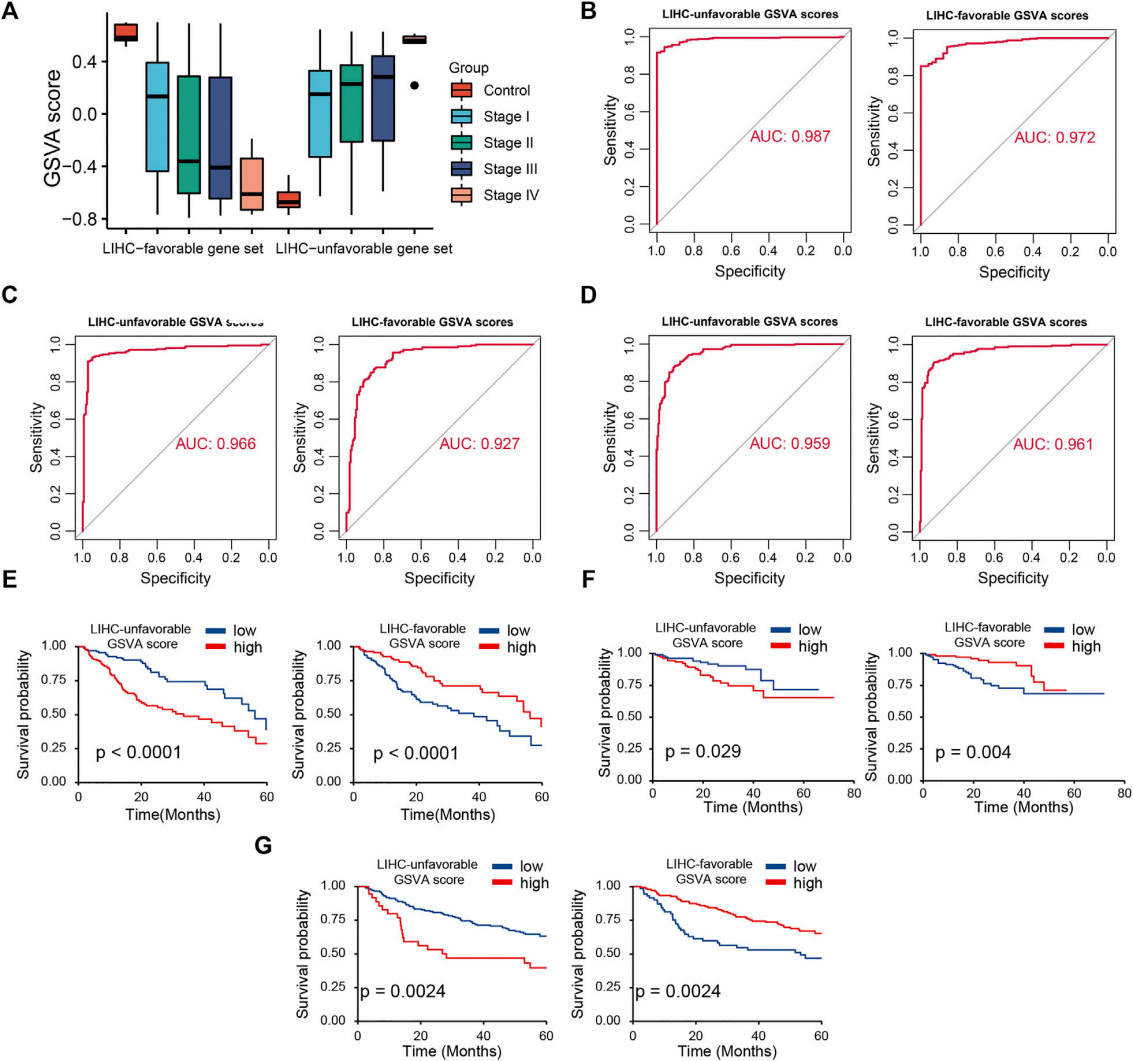
Diagnostic and prognostic abilities of LIHC-unfavorable GSVA score (LUG score) and LIHC-favorable GSVA score (LFG score). **(A)** Box plot of LUG score and LFG score in different LIHC stages. **(B–D)** ROC curves analysis of LUG score and LFG score in TCGA, ICGC and GSE14520. **(E–G)** Survival analysis of LUG score and LFG score in TCGA, ICGC and GSE14520.

**FIGURE 5:**
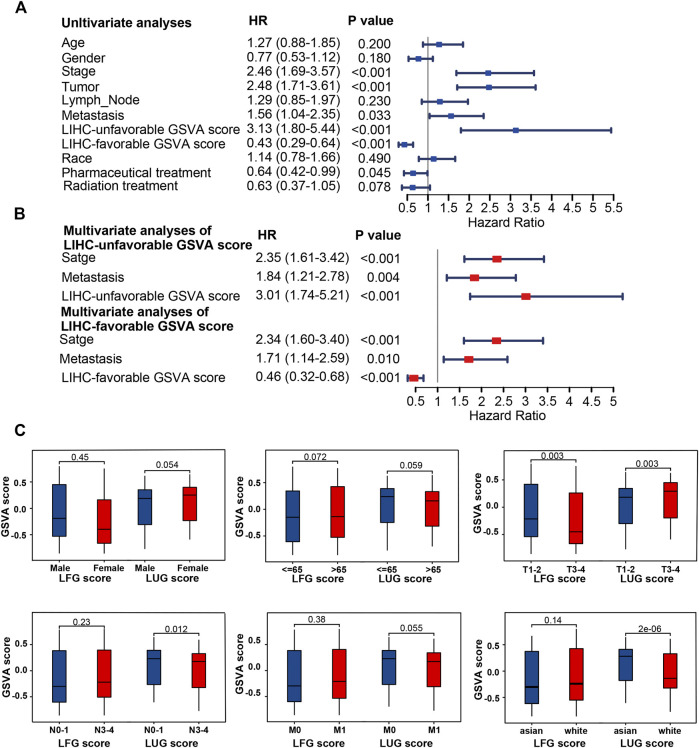
Clinical correlation analyses and of LUG score and LFG score. **(A,B)** Univariate and multivariate Cox regression analysis. **(C)** Correlation of LUG score and LFG score with clinical features.

Based on two prognosis-related gene sets (LUGs and LFGs), we used GSVA algorithm to construct two LIHC-progression GSVA scores, named LIHC-unfavorable GSVA score (LUG score) and LIHC-favorable GSVA score (LFG score) respectively. Obviously, the LUG score gradually increased as the tumor progresses in HCC patients, while the LFG score was complete opposite (Figure 4A). ROC analysis proved that both LUGs and LFGs had great diagnostic accuracy in diverse independent verification datasets, among which AUC = 0.987 and 0.972 in TCGA, AUC = 0.966 and 0.927 in GSE14520, and AUC = 0.959 and 0.961 in ICGC (Figures 4B–D). As shown in Figures 4E–G, survival analyses indicated patients from the low-LFG score group or high-LFG score group had a longer OS than those from the high-LFG score group or high-LFG score group. According to the univariate/multivariate Cox regression analysis, TNM stage, LUG score, and LFG score can serve as independent predictors to evaluate the prognosis of HCC patients (Figures 5A,B). Subsequently, we explored the relevance between the GSVA scores and other clinicopathological parameters. The result indicated the LFG score was significantly related to T stage, and the LUG score has a marked correlation with T stage, N stage, and race (Figure 5C).

### Genetic and Transcriptional Alterations of GSVA Scores and LCGs in LIHC

**FIGURE 6:**
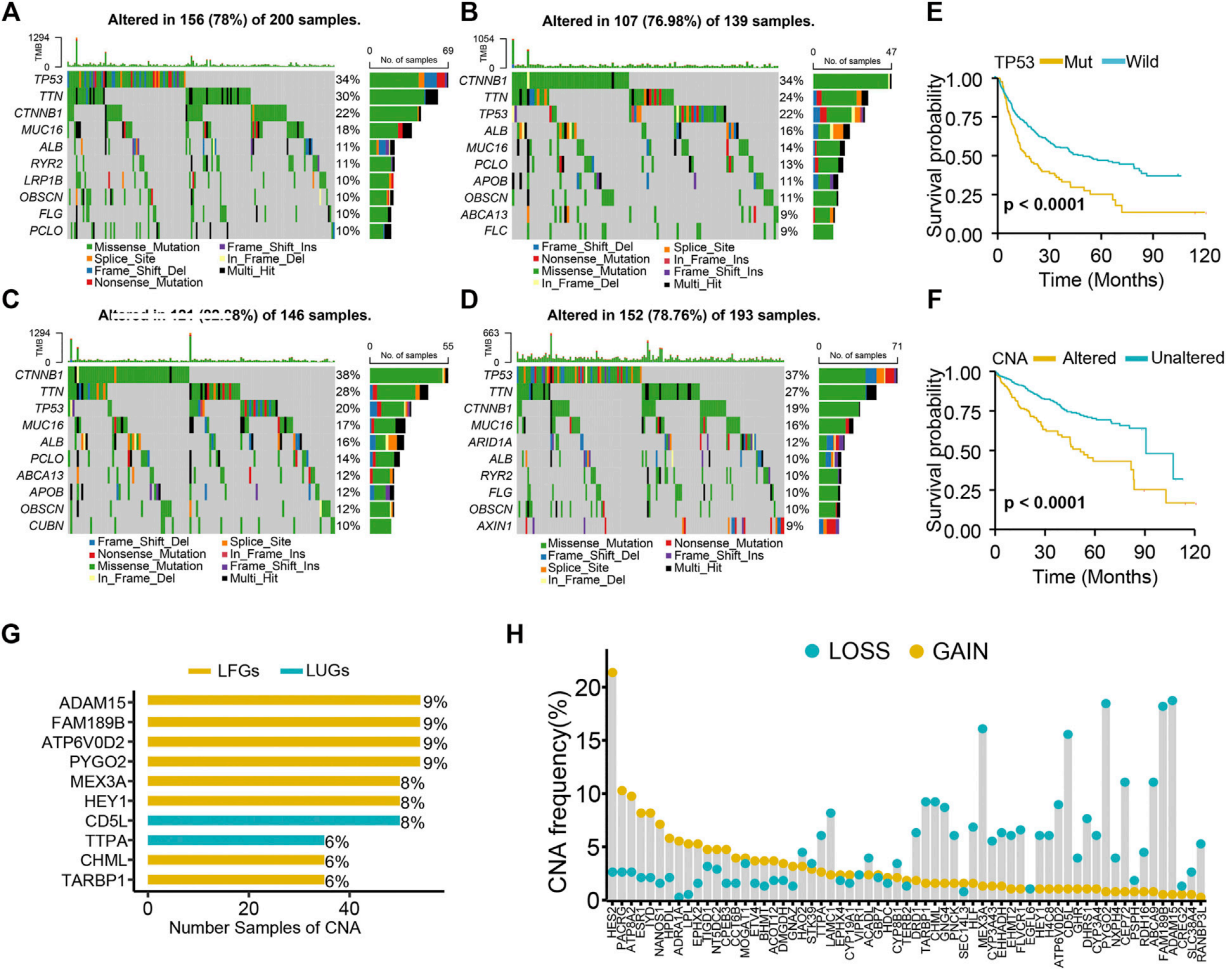
Genetic alteration analysis. **(A)** Mutation landscape of high-LUGs score group, **(B)** low-LUGs score group, **(C)** high-LFGs score group, and **(D)** low-LFGs score group. **(E)** KM curve of TP53 mutation. **(F)** KM curve of CNA. **(G)** The top 10 LCGs with the highest frequency of CNV. **(H)** Frequencies of CNV gain, loss, and non-CNV among LCGs.

Both the high-LUG score group and low-LFG score group had a higher TP53 mutation rate than the low-score groups (Figures 6A–D). The prognosis of patients with TP53 mutations was significantly worse than those with wild TP53 (Figure 6E). Because of the high mutation frequency and poor prognostic feature of TP53, we evaluated the relationship between TP53 mutation and LCGs expression. The results showed that the expression levels of 21 of the 63 LCGs were significantly associated with TP53 mutation status (Supplementary Figure S3).

We found high CNA frequency in patients who seemed to presage poor prognosis (Figure 6F) and prevalent copy number alterations in all LCGs (Figure 6G). LCGs with CNV gain, such as NT5DC2, GNAZ, and HPDL, were significantly elevated in LIHC samples, while LCGs with CNV loss, such as CYP3A4, GHR, and HLF, were decreased in LIHC samples, suggesting that CNV might regulate the mRNA expression of LCGs (Figure 6H). However, some LCGs with CNV loss, such as EHMT2 and HEY1, showed upregulated expression, while other LCGs with abnormal expression showed no differences of frequency between CNV gain and loss. Hence, although CNV can explain expression variation in many LCGs, CNV is not the only factor involved in the regulation of mRNA expression (Sebestyen et al., 2016).

### Potential Molecular Mechanism of Two GSVA Scores

**FIGURE 7:**
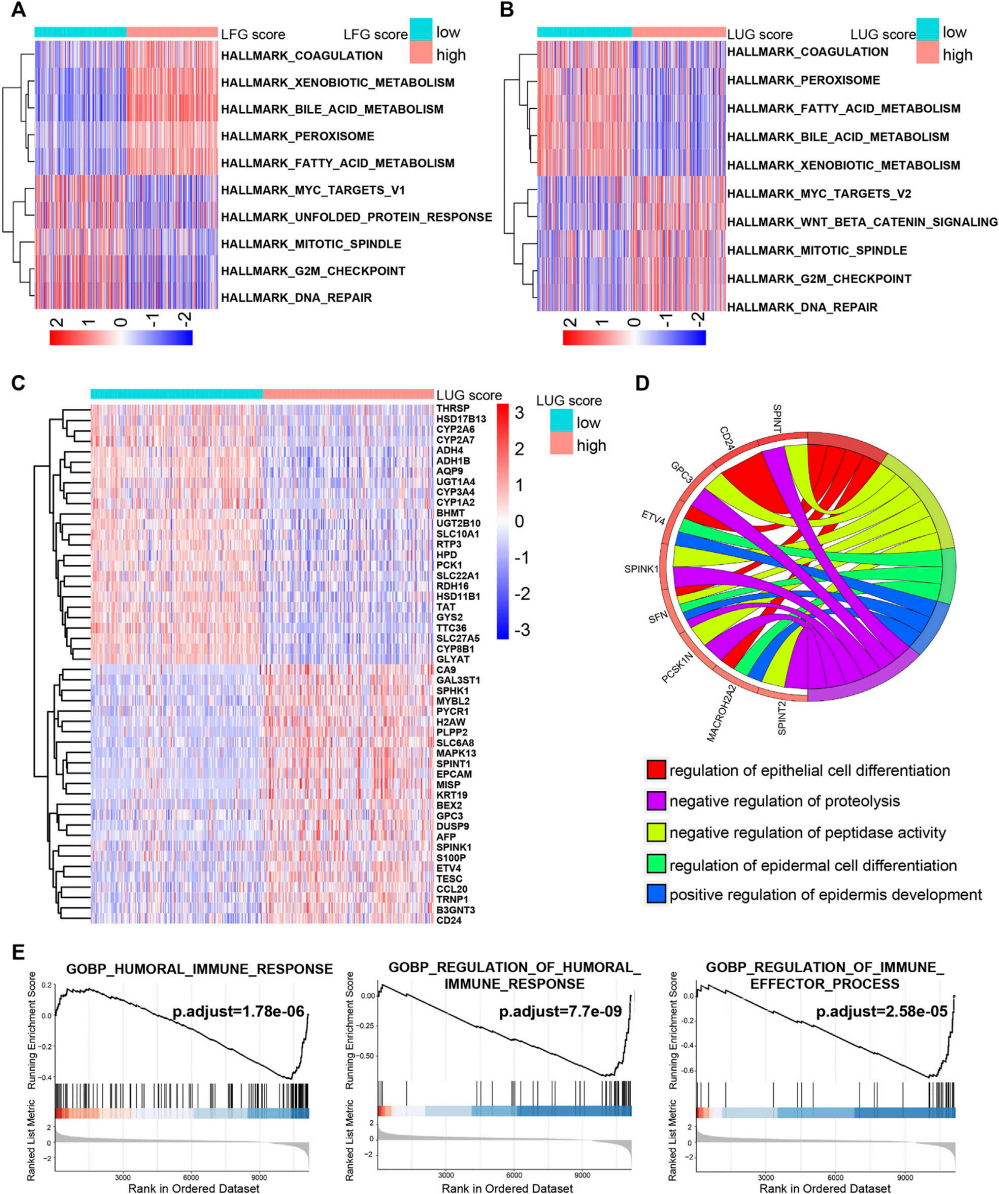
The potential molecular mechanism of the prognostic score. **(A**,**B)** GSVA-HALLMARK for LFG score and LUG score. **(C)** The heatmap of DEGs between high- and low-LUG score groups. **(D)** GO function annotation of DEGs. **(E)** GSEA using immune gene set.

Both the high-LFG score group and low-LUG score group were significantly enriched for metabolisms, such as fatty acid metabolism, bile acid metabolism, and xenobiotic metabolism, while the activity of pathways related to cell cycle, such as G2M checkpoint, mitotic spindle, and DNA repair mitotic spindle enriched significantly in the low-LFG score group and high-LUG score group (Figures 7A,B). GO enriched the annotation of upregulated DEGs in the high-LUG score group showed the significant activated functional pathways related to cell differentiation, including differentiation regulation of the epidermal cell and epithelial cell (Figures 7C,D). It is worth noting that the immune responses were mainly active in the high-LUG score group, as revealed by GSEA (Figure 7E).

### Four Key LCGs Were Screened out by PPI Network Analysis and Univariate Cox Regression Method

**FIGURE 8:**
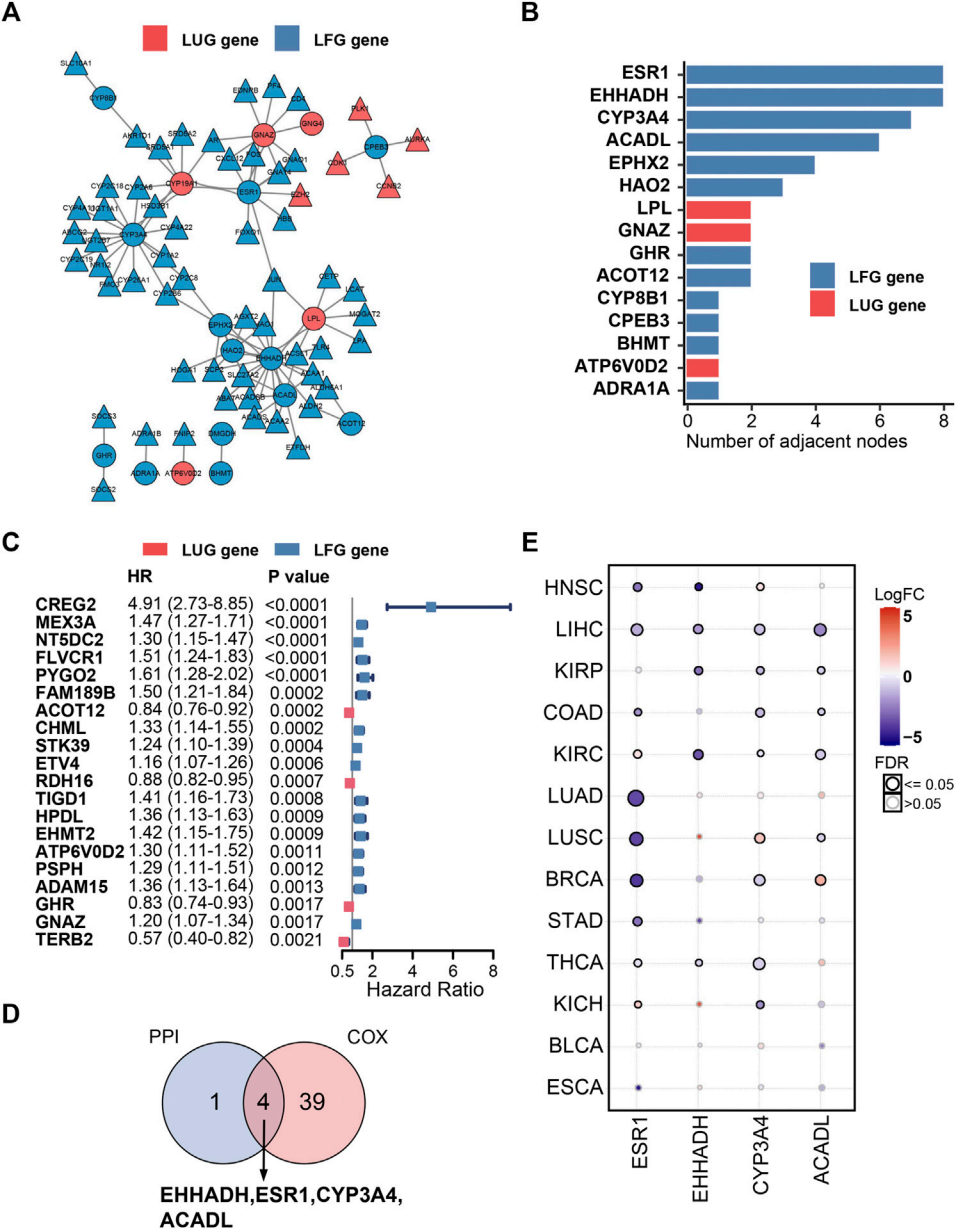
PPI network and univariate Cox regression analysis. **(A)** PPI network of the LDGs. **(B)** The top 15 genes ordered by the number of nodes. **(C)** Univariate Cox regression analysis of LCGs. **(D)** Venn diagram displaying the key LCGs. **(E)** Pan-cancer analysis of key LCGs from GSCA database.

**FIGURE 9:**
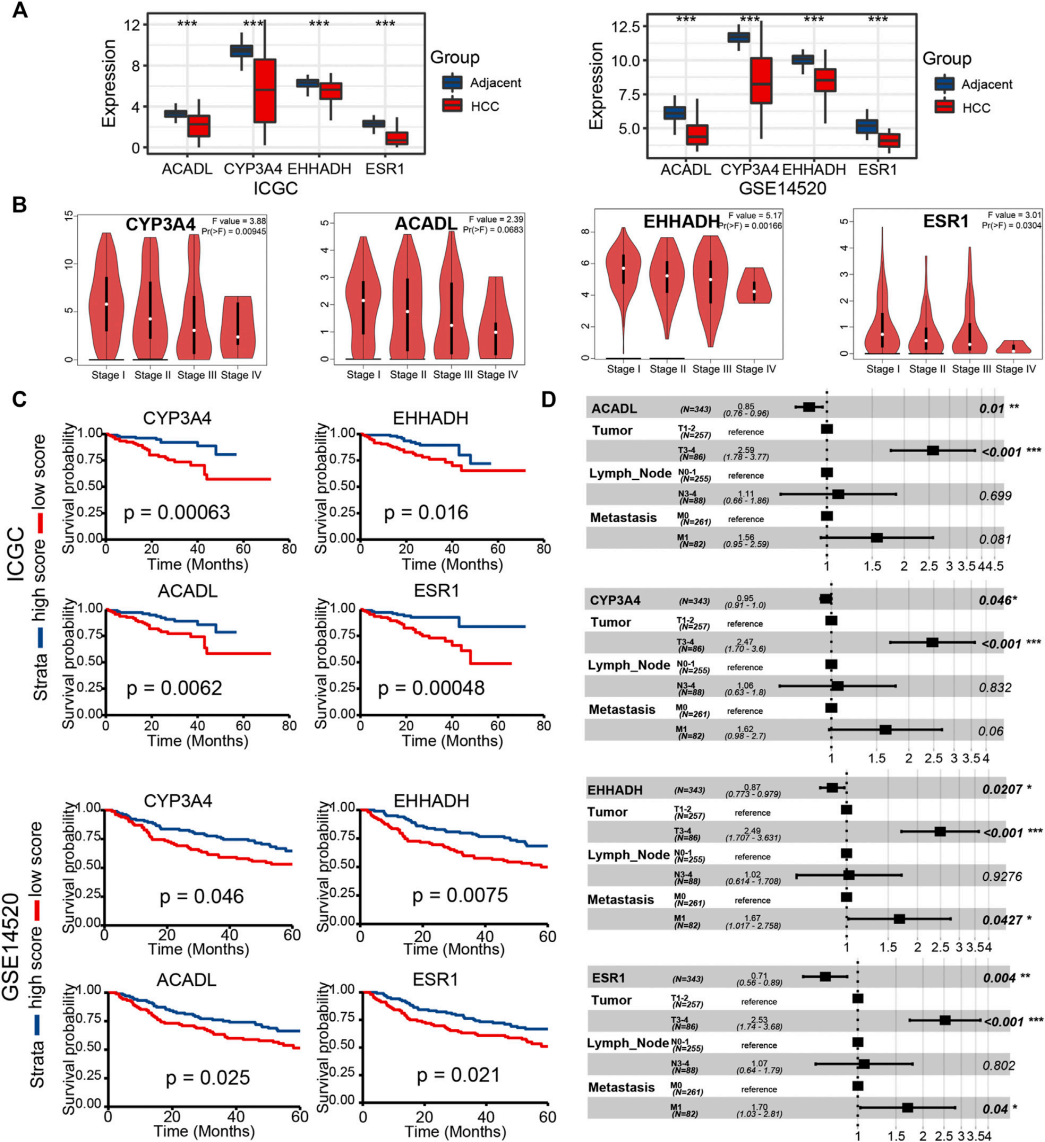
Validation of key LCGs in expression level and prognostic signification **(A)** The expression level of key genes in LIHC tissues and normal tissues based on ICGC and GSE14520. **(B)** Differentiated expression of key genes in different LIHC stages based on the GEPIA database. **(C)** KM plots of key LCGs based on ICGC and GSE14520. **(D)** Multivariate Cox regression of key LCGs. *p < 0.05, **p < 0.01, ***p < 0.001, ****p < 0.0001.

A PPI network, composed of 77 nodes and 152 edges, was built using the STRING database (Figure 8A). As shown in Figure 8B, the importance of LCGs was ordered by their number of adjacent nodes in the network. On the other hand, a total of 40 LCGs could affect the outcome of HCC patients according to univariate Cox regression analysis (Figure 8C). Eventually, four LFGs (ESR1, EHHADH, CYP3A4, and ACADL) were selected as the key LCGs by integrated analysis of survival evaluation and PPI network (Figure 9D). Pan-cancer research indicated these four key LCGs also apparently decreased in a variety of cancers (Figure 8E).

### Validation of Key LCGs Expression and Prognosis in External Data

In the two external datasets (ICGC and GSE14520), gene expression levels of four key LCGs were also lower in the liver cancer tissue than adjacent tissue, which is in line with previous researches (Figure 9A). Moreover, the box plot of gene expression at different stages obtained from GEPIA proved that four key LCGs possessed similar expression patterns in the HCC progression (Figure 9B). KM survival curves based on ICGC and GSE14520 cohorts demonstrated key LCGs performed great efficiency for distinguishing prognostic different HCC patients (Figure 9C). Combining the clinical features with the key LCGs expression, multivariate Cox regression validated that the key LCGs were independent prognostic factors and protective factors (Figure 9D; Supplementary Figure S4). Pathway analyses of GSCA showed that all key LCGs might participate in Hormone pathways, and EHHADH is probably connected with the RTK pathway (Supplementary Figure S5A). Meanwhile, the methylation level of ESR1 and CYP3A4 in tumor samples was significantly higher than that in normal samples, implying that methylation could be one of the factors leading to abnormal gene expression (Supplementary Figure S5B). Single gene enrichment analysis based on HCCDB and Metascape revealed that the key LCGs had remarkable correlation with metabolism pathways, which was in keeping with previous results on GSVA scores (Supplementary Figure S6).

### Immune Infiltration and Drug Susceptibility Analysis

**FIGURE 10:**
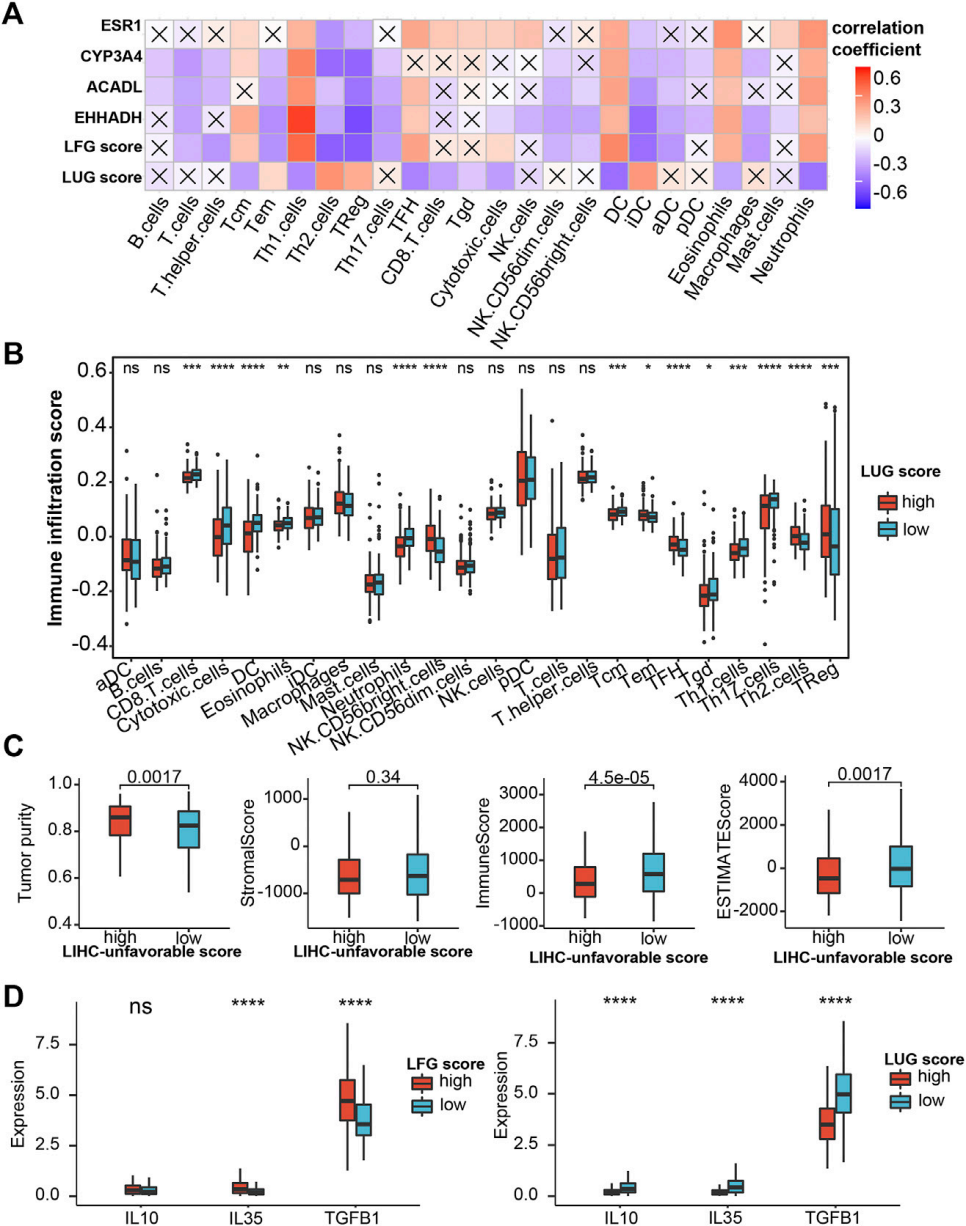
Evaluation of immune infiltration **(A)** Correlation heatmap of LFG score, LUG score and key LCGs with 24 immune cells. The cross indicates no significance. **(B)** Immune infiltration score in the high- and low-LUG score group. **(C)** Correlations of LUG score with immune score, stromal score, ESTIMATEScore and tumor purity. **(D)** Expression of the immune suppressive cytokines between high- and low-LUG/LFG score group. *p < 0.05, **p < 0.01, ***p < 0.001, ****p < 0.0001.

We performed the ssGSEA algorithm to assess the association of the abundance of immune cells with two GSVA scores and the key LCGs. As shown in Figures 10A,B, the LFGs score similar to the key LCGs, were positively correlated with Th1 cells, DC, Eosinophils and Neutrophils, while negatively correlated withTh2 cells, TReg and iDC. Interestingly, the LFGs score performed oppositely in these immune cells compared with the LFGs score (Figures 10A,B). Tumor purity in the high-LFG score group was significantly higher than those in the low-LFG score group, and StromalScore, ImmuneScore, and ESTIMATEScore in the low-LFG score group were significantly higher than those in the high-LFG score group (Figure 10C). Chemokines involved in the immunosuppressive process induced by Tregs (IL-4, IL-35, and TGF-β) were also significantly upregulated in the high-LUG score group and low-LFG score group.

### Immunotherapy Response and Drug Susceptibility Analysis

**FIGURE 11:**
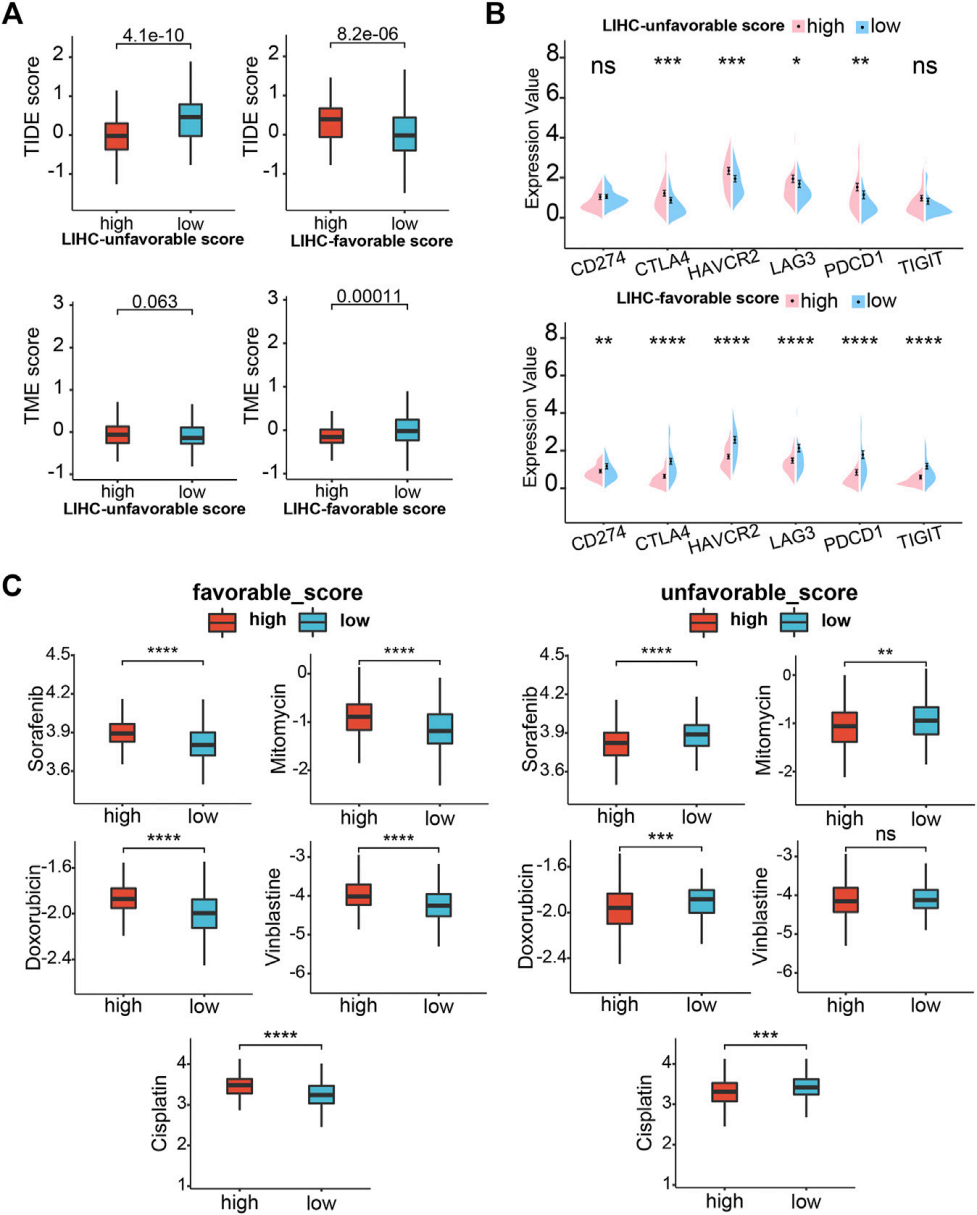
Drug sensitivity analysis. **(A)** TIDE scores and TME score between high- and low-LUG/LFG score group. **(B)** Correlation analysis of two GSVA scores and six immune checkpoint genes. **(C)** Relationships between chemotherapeutic sensitivity and both LFG score and LUG score. *p < 0.05, **p < 0.01, ***p < 0.001, ****p < 0.0001.

Subsequently, we analyzed the correlation between GSVA scores and multiple immunotherapy response-related indices to assess their impacts on immunotherapy. Patients with low-LUG scores get a higher TIDE score and lower TME score than those with high-LUG score (Figure 11A). In addition, we investigated the associations between immune checkpoints and our GSVA scores. Figure 10E shows that several immune checkpoints were differentially expressed in the two groups, including PD-1, PD-L1, and CTLA-4 (Figure 11B). These results demonstrated that patients with low LFG scores tended to have a better immunotherapy response. We next selected chemotherapy drugs recommended for liver cancer treatment by AJCC guidelines to evaluate the sensitivities of patients in the low- and high-GSVA score groups to these drugs. Interestingly, we found that the patients in the high-LUG score group or low-LFG score group had lower IC50 values for Sorafenib, Doxorubicin, Doxorubicin, and Cisplatin. Together, these results showed that LUG score and LFG score were related to drug sensitivity (Figure 11C).

## Discussion

LIHC is a common digestive system tumor with high aggressiveness and poor prognosis. LIHC is insensitive to conventional radiotherapy and chemotherapies; consequently, surgery becomes the main treatment (Novikova et al., 2017). Unfortunately, only 30%–40% of LIHC patients are eligible for surgical resection, and the recurrence rate after surgery is very high (Cao et al., 2012). Therefore, it is urgently needed to explore reliable biomarkers that can be regarded as potential diagnostic and therapeutic targets.

With the rapid progress and widespread application of high-throughput sequencing technology, integrated bioinformatics analysis has emerged as a promising approach to explore various diagnostic and prognostic biomarkers for different tumors. In our research, LIHC data from TCGA were used for bioinformatics analysis to identify genes that were differentially expressed in different stages. Interestingly, we found gene expression patterns of some DEGs incrementally or digressively changed with LIHC development. For example, a gene may be obviously differentially expressed in the advanced stage but not in the early stage. Thus, we considered these LIHC-development genes may have an impact on cell malignant transformation and tumor evolution. With tumor deterioration, there were 330 LDGs screened out, including 83 LUGs gradually upregulated and 247 LFGs gradually downregulated. Additionally, GO functional enrichment analysis indicated that LDGs were significantly involved in the regulation of immunity. Results from KEGG pathway enrichment analysis manifested LDGs were enriched for the chemical carcinogenesis and PPAR signaling pathway.

After considering the prognostic factors, the number of LDGs was further reduced to 31 LUGs and 32 LFGs. It has been reported that certain LUGs and LFGs are related to the formation and regulation of tumor progression. EMHT2 encodes a methyltransferase that is significantly associated with HCC progression and aggression (Wei et al., 2017). CHML promotes HCC metastasis and leads to poor survival, early recurrence, and more satellite nodules (Chen et al., 2019). STK39 contributes to the progression of HCC by the PLK1/ERK signaling pathway (Zhang et al., 2021). ARID2 expression significantly decreased in metastatic HCC tissues, showing a negative correlation with pathological grade and organ metastasis, and a positive association with survival of HCC patients (Jiang et al., 2020). These results confirmed the possibility that LUGs and LFGs can be used as a prognostic model for LIHC.

In the previous studies, it is the common way that a gene set is analyzed by Cox regression and every gene can get a coefficient that can construct the prognostic model. Nevertheless, because of the heterogeneity of the tumor and the limitations of the sample size, the coefficient of a gene is almost impossible to determine. Thus, we took advantage of GSVA methods to calculate individual samples’ NES as prognostic features based on LUGs and LFGs. ROC curve analysis and KM analysis suggested the two GSVA scores had precise diagnosis and prognosis capacity, which were verified in the other two independent LIHC datasets. Univariate and multivariate Cox regression analysis also substantiated that LUG score and LFG score were independent prognostic factors for LIHC.

Four LCGs (ESR1, EHHADH, CYP3A4, and ACADL) were identified as key prognosis-related LCGs based on a combination of the PPI network and univariate Cox regression analysis. CYP3A4 encodes a member of the cytochrome P450 superfamily of enzymes and can influent the chemoresistance of LIHC thus leading to a poor prognosis (Ashida et al., 2017). ESR1 has been a focus in breast cancer, and its mutation is a common cause of acquired resistance (Dustin et al., 2019). ACADL restrains hepatocellular carcinoma by targeting Hippo/YAP signaling (Zhao et al., 2020). We have reason to believe the potential effects of these genes to LIHC, although exploration is still insufficient now.

Through the research on the molecular mechanism of prognostic signatures and score models, we found that the high-LFG score group with a poor prognosis was remarkably enriched in the active metabolism, while the high-LUG score group with a poor prognosis not only exhibited low immune response and metabolic activity but also involved cell cycle regulation. The key LCGs belonged to protective factors and were involved in the metabolic process in HCC. Active metabolism was considered as one of the important signatures of a good prognosis of HCC (Yang et al., 2020; Liu et al., 2021c).

As a continuous breakthrough in the field of immunotherapy, emerging research shows that the tumor microenvironment can regulate cancer progression (Hinshaw and Shevde 2019). Increasing evidence shows that LIHC tissue is often infiltrated by many types of Immune cells (Ringelhan et al., 2018). Th1 cells participate in effective anti-cancer response but Th2 cells show a low cytolytic and antigen-presenting activity. Increase of T2 cells and decrease of T1 cells in intra-tumor are inversely associated with HCC patient survival (Foerster et al., 2018). Our research showed Th2 cells were significantly reduced in the high-LUG score group with a poor prognosis.

DCs play a key role in the initiation and regulation of the immune response. Mature DCs can guide the body to produce a specific immune response and play an anti-tumor role. On the other hand, immature DCs can lead to immune tolerance by activating the body to produce regulatory T cells, anergic T cells, or tolerant T cells (Dhodapkar et al., 2001). In this study, we found that high infiltration of immature DCs mainly happened in the high-LUG score group, while LUG score was negative with infiltration of Mature DCs.

Tregs can promote immunosuppression via secreting immune suppressive cytokines (IL-10, IL-35, TGF-β) or expressing co-inhibitory molecules such as CTLA-4, PD-1, LAG-3, and TIGIT (Josefowicz et al., 2012; Kumar et al., 2018). In the present study, Tregs are upregulated in the high-LUG score group and low-LFG score group. Additionally, cytokines (IL-10, IL-35, TGF-β) related to the immunosuppression process and co-inhibitory checkpoints (CTLA-4, PD-1, and LAG-3) were all upregulated in the high-LUGs score group, which validated that the immunosuppression induced by Tregs exists in high-LUGs score tumors.

Immune checkpoint inhibitors can block immune checkpoints on the cell membrane, which become a promising strategy in the treatment of cancer. Although a variety of immune checkpoint inhibitors has been widely applied in the front-line treatment of HCC, many advanced LIHC patients are resistant to immune checkpoint therapy (Donisi et al., 2020). Our study reveals multiple immune checkpoints (like PD-1, PD-L1, and CTLA4) expression upregulated in high-risk groups. Low TIDE score and high TME score mean a high probability of response to immune checkpoint blockade therapy. We observed that TME scores were significantly higher in high-LFG score groups than those with low-LFG score groups and TME score is completely opposite. Meanwhile, patients with low LFG scores had high expression of multiple immune checkpoints (CTLA4, CD247, HAVCR2, LAG3, PDCD1, and TIGIT). Therefore, we estimate the LFG score possibly can predict the response of immune checkpoint therapy, and combined immunotherapy may be a better choice for the treatment of LIHC.

Nonetheless, several limitations were notable in our study. First, since all data were collected retrospectively, the potential bias of clinicopathological features is inevitable. Second, the two gene sets may be too large to economize on the sequencing costs. Finally, large-scale prospective studies and functional and mechanistic experimental studies are needed to support our findings.

## Conclusion

In summary, we discover two LIHC-progression characteristic gene sets and created two LIHC-progression GSVA scores with great diagnostic and prognostic values for hepatocellular carcinoma. Our findings are of great importance in developing new prognostic markers and molecular targets for LIHC.

## Data Availability

The datasets presented in this study can be found in online repositories. The names of the repository/repositories and accession number(s) can be found in the article/Supplementary Material.
