# Supplementary figures and images for: Construction and Validation of Two Hepatocellular Carcinoma-Progression Prognostic Scores Based on Gene Set Variation Analysis
Source: Front Cell Dev Biol. 2022 Mar 9;10:806989. doi: 10.3389/fcell.2022.806989 (PMC8959467; doi:10.3389/fcell.2022.806989)

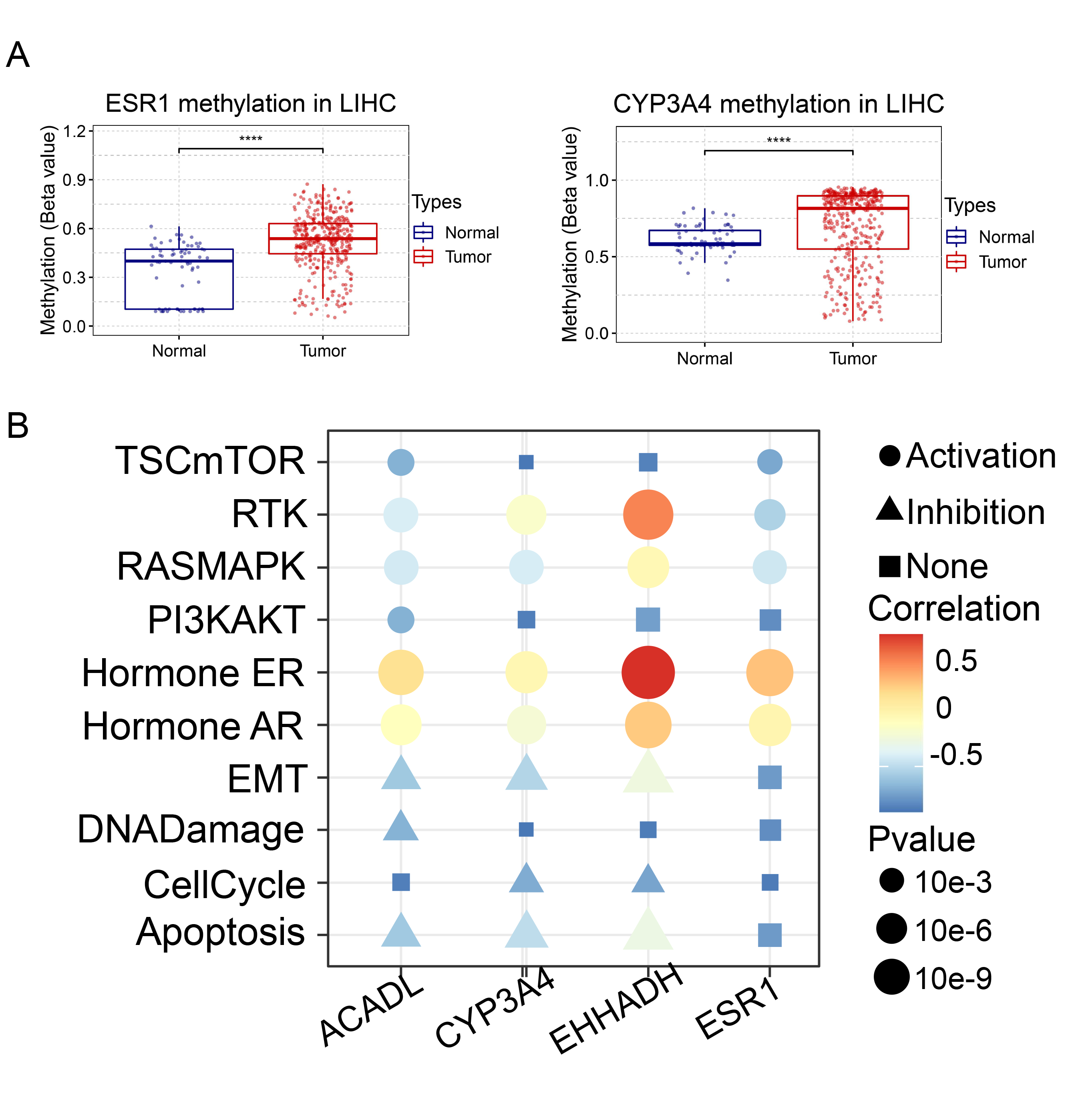

Supplement: Supplementary file 1 [file Image6.TIF]

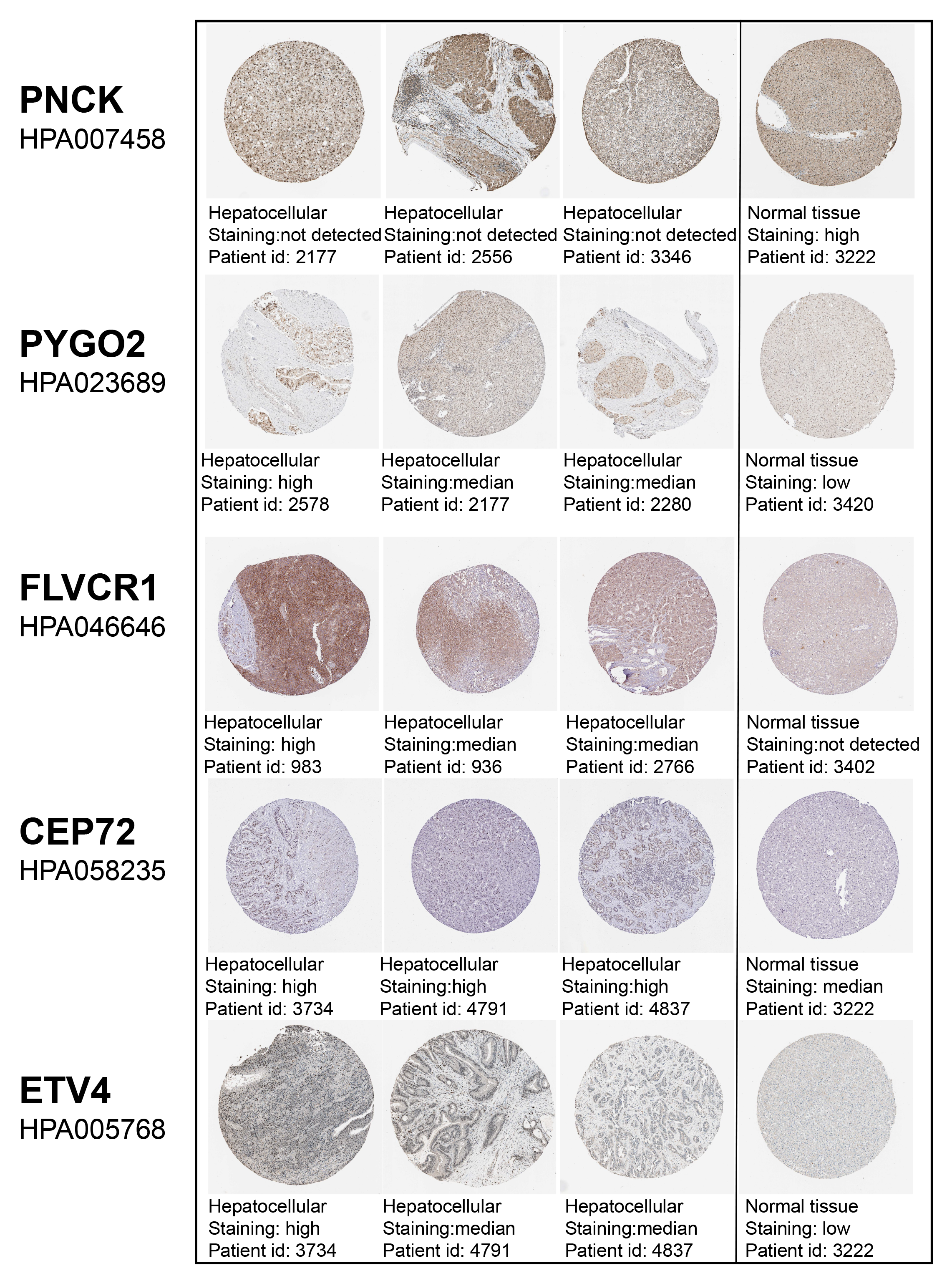

Supplement: Supplementary file 2 [file Image3.TIF]

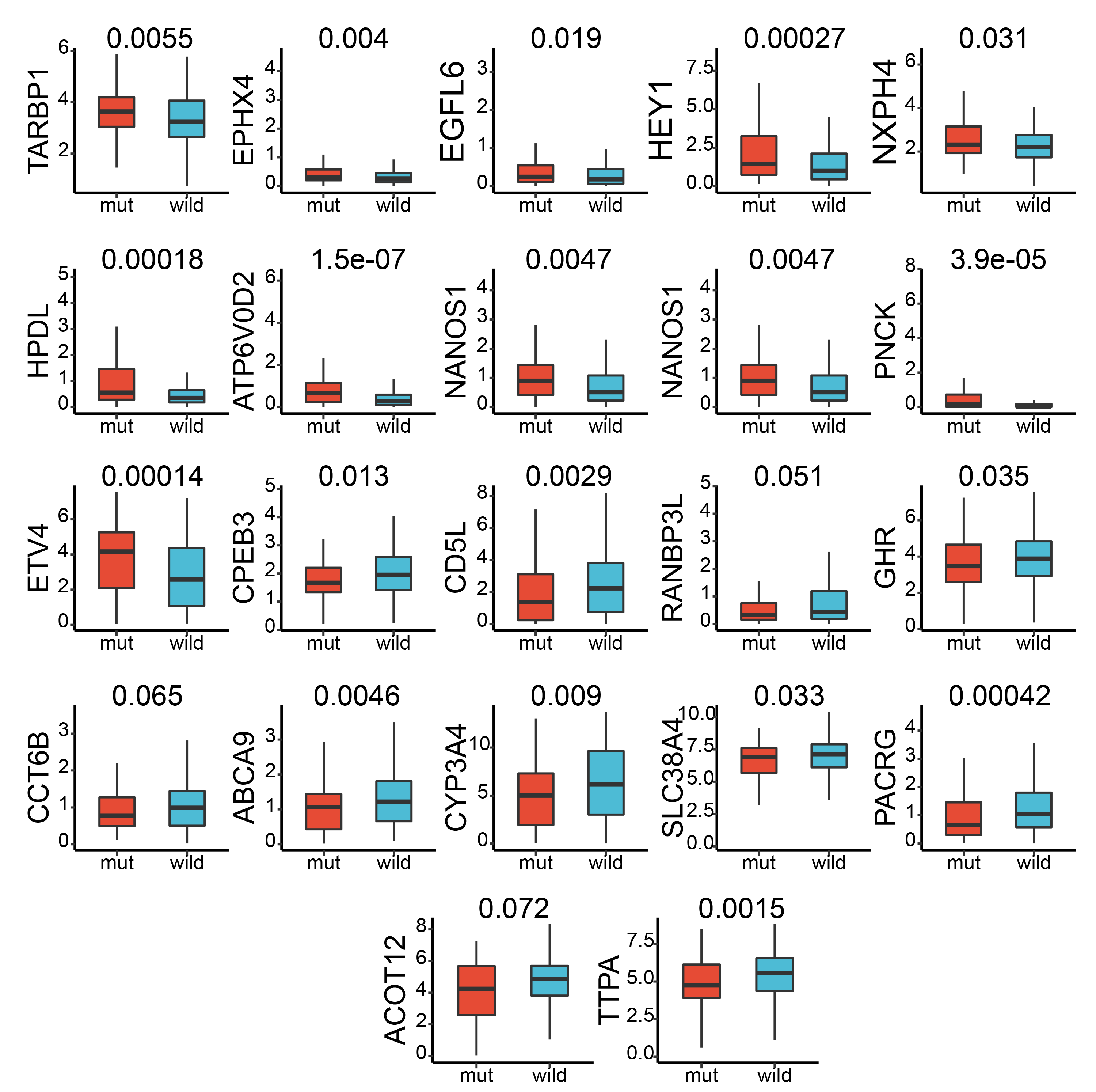

Supplement: Supplementary file 3 [file Image4.TIF]

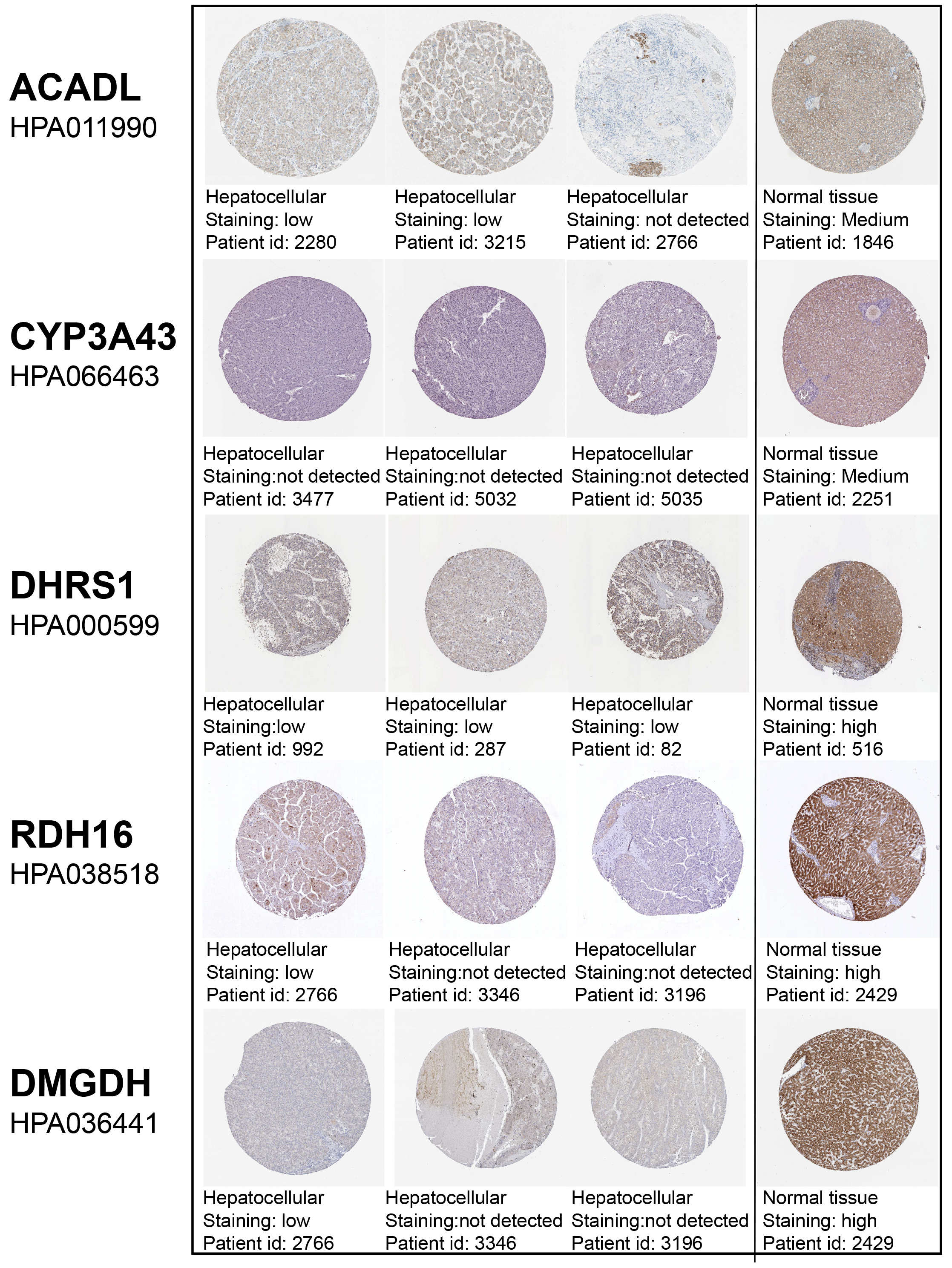

Supplement: Supplementary file 4 [file Image2.TIF]

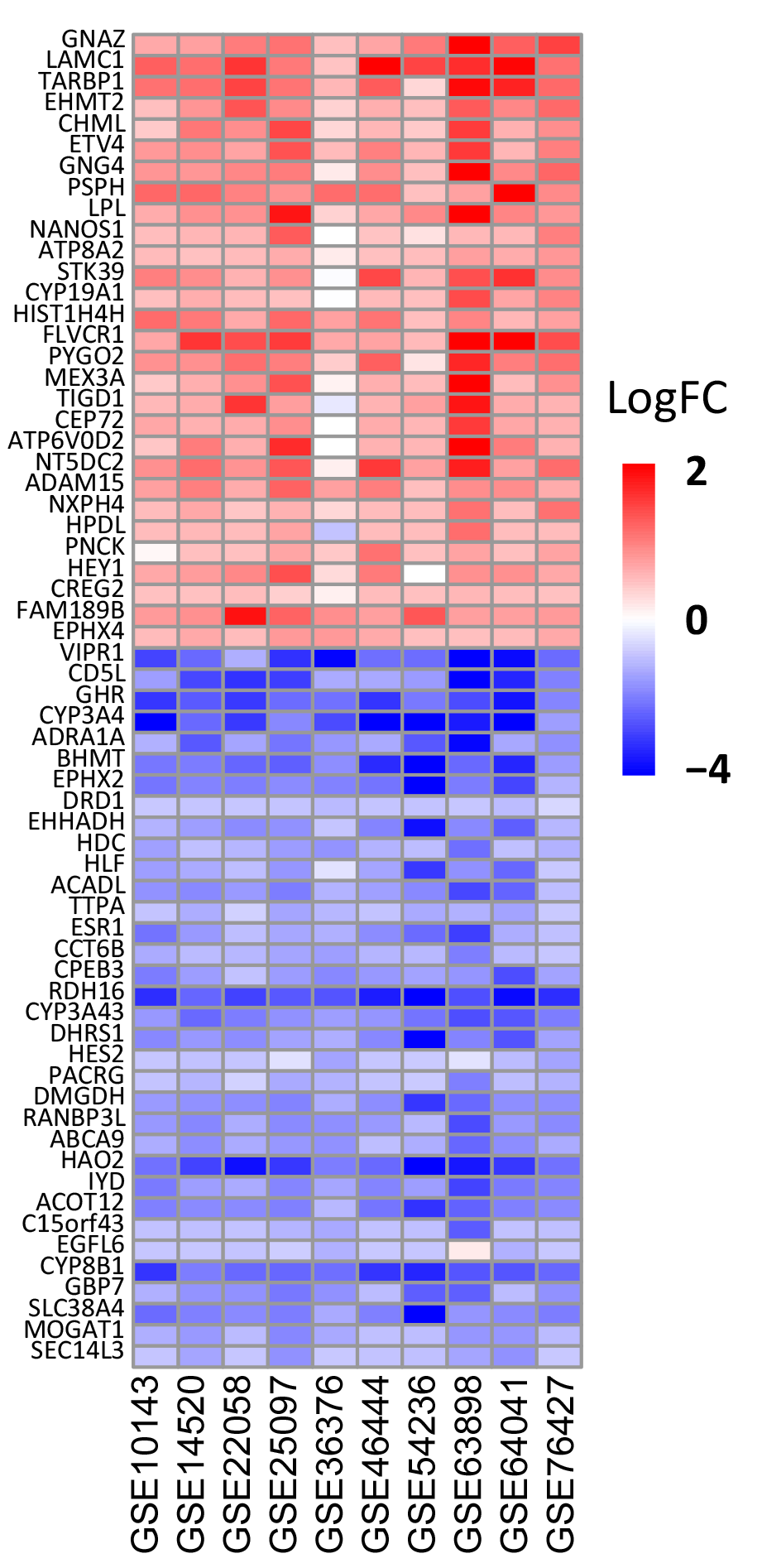

Supplement: Supplementary file 5 [file Image1.TIF]

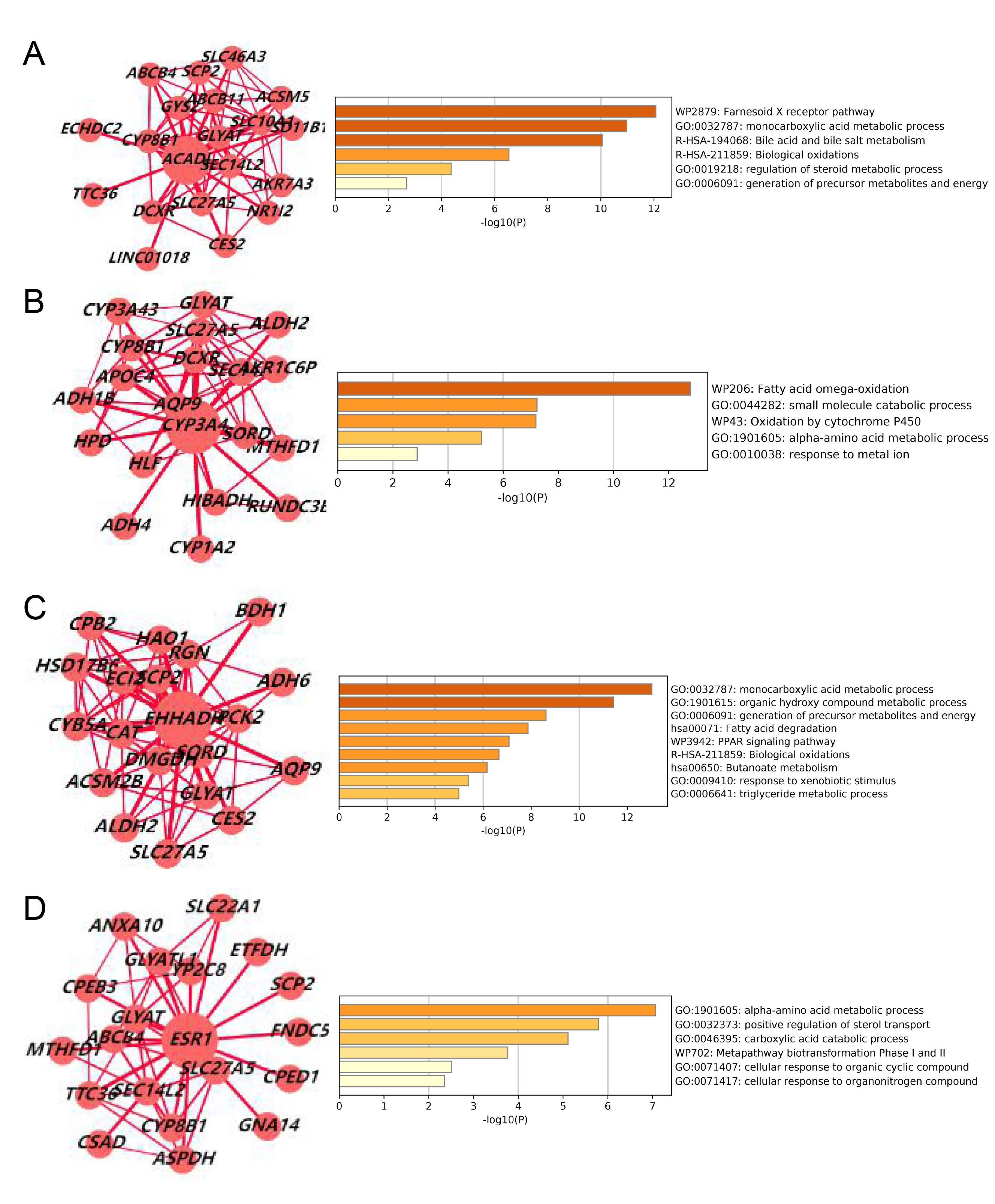

Supplement: Supplementary file 6 [file Image7.TIF]

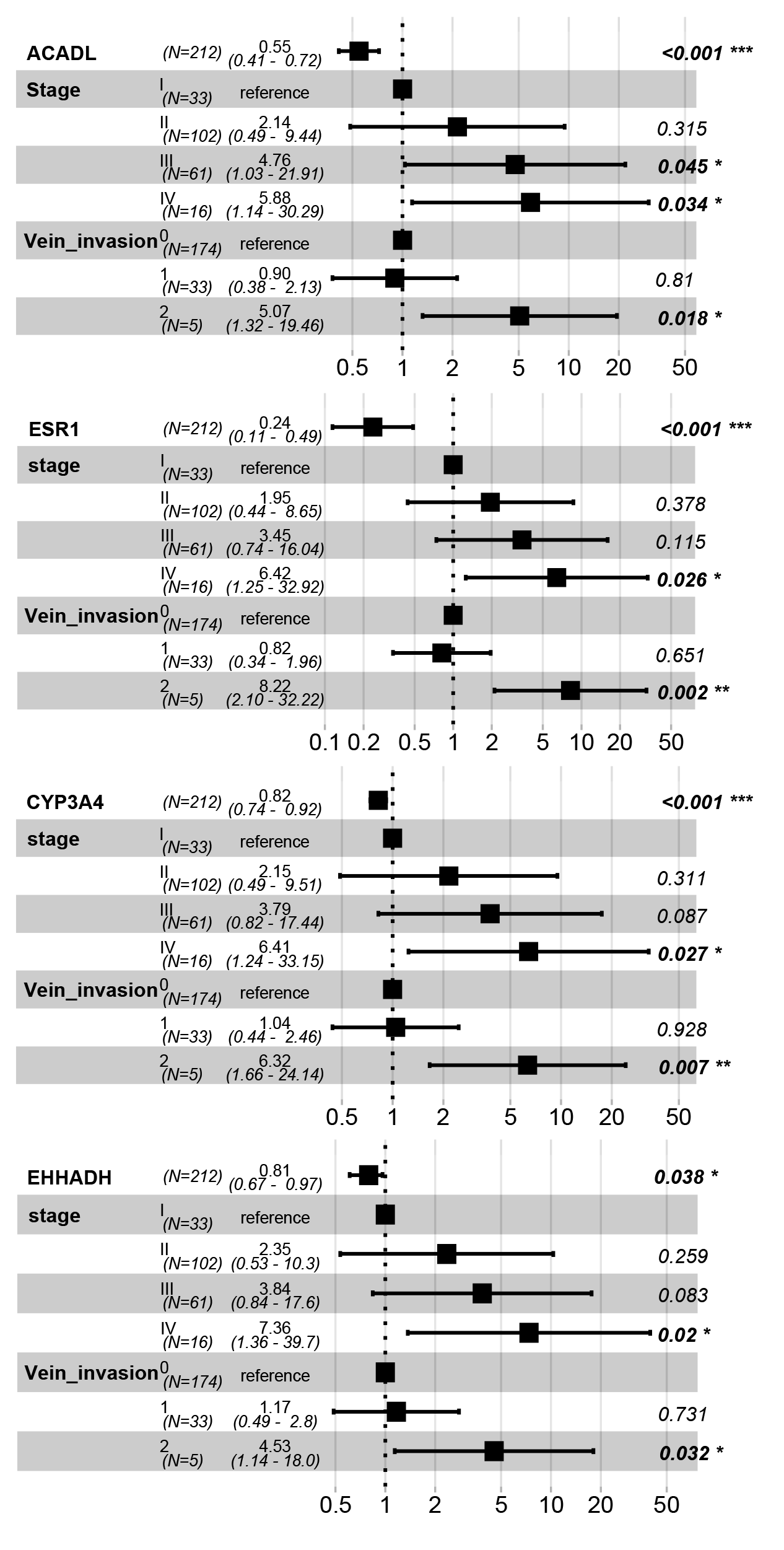

Supplement: Supplementary file 7 [file Image5.TIF]
